# What US Cardiology Can Learn From the 2023 ESC Guidelines for the Management of Acute Coronary Syndromes

**DOI:** 10.1002/clc.24329

**Published:** 2024-08-12

**Authors:** Nanette K. Wenger

**Affiliations:** ^1^ Consultant, Emory Heart and Vascular Center, Founding Consultant, Emory Women's Heart Center Emory University School of Medicine Atlanta Georgia USA

## 1 Introduction

Recognizing that acute coronary syndromes (ACSs) constitute a spectrum encompassing unstable angina, non‐ST elevation myocardial infarction (NSTEMI), and ST elevation myocardial infarction (STEMI), the 2023 European Society of Cardiology Guidelines [1] for the management of ACSs addressed all three. This differs from the prior US guidelines that individually addressed unstable angina, NSTEMI, and STEMI. The 2023 ESCACS Guidelines thus encompass comprehensive patient management from admission to long‐term care, again including what in prior US guidelines would have been a secondary prevention guideline. In addition, the task force included a patient member who provided a patient perspective that is highlighted in the European publication.

## 2 Chest Pain

Because 80% of both women and men with ACS present with chest pain or pressure, this symptom is detailed in the guidelines, derived in part from the US Chest Pain guideline [2]. The ESC document noted that additional symptoms such as diaphoresis, indigestion or epigastric pain, and shoulder or arm pain occur commonly in both women and men with an ACS. However, some symptoms appear to be more common in women, including dizziness and syncope, nausea and vomiting, jaw and back pain, shortness of breath, pain between the shoulder blades, palpitations, and fatigue.

## 3 The ACS Spectrum

In advancing the science and implementation, the 2023 Guidelines offer a conceptual approach of five items: think A.C.S. at the initial assessment, think invasive management, think antithrombotic therapy, think revascularization, and think secondary prevention. To further explain the thinking A.C.S. at the initial evaluation of patients with suspected ACS, “A” relates to an abnormal ECG (performing an ECG urgently to assess for evidence of ischemia or other abnormalities), “C” considers the clinical context and other available investigations, and “S” for stable, performing an examination to assess whether the patient is clinically and vitally stable.

**Figure 1:**
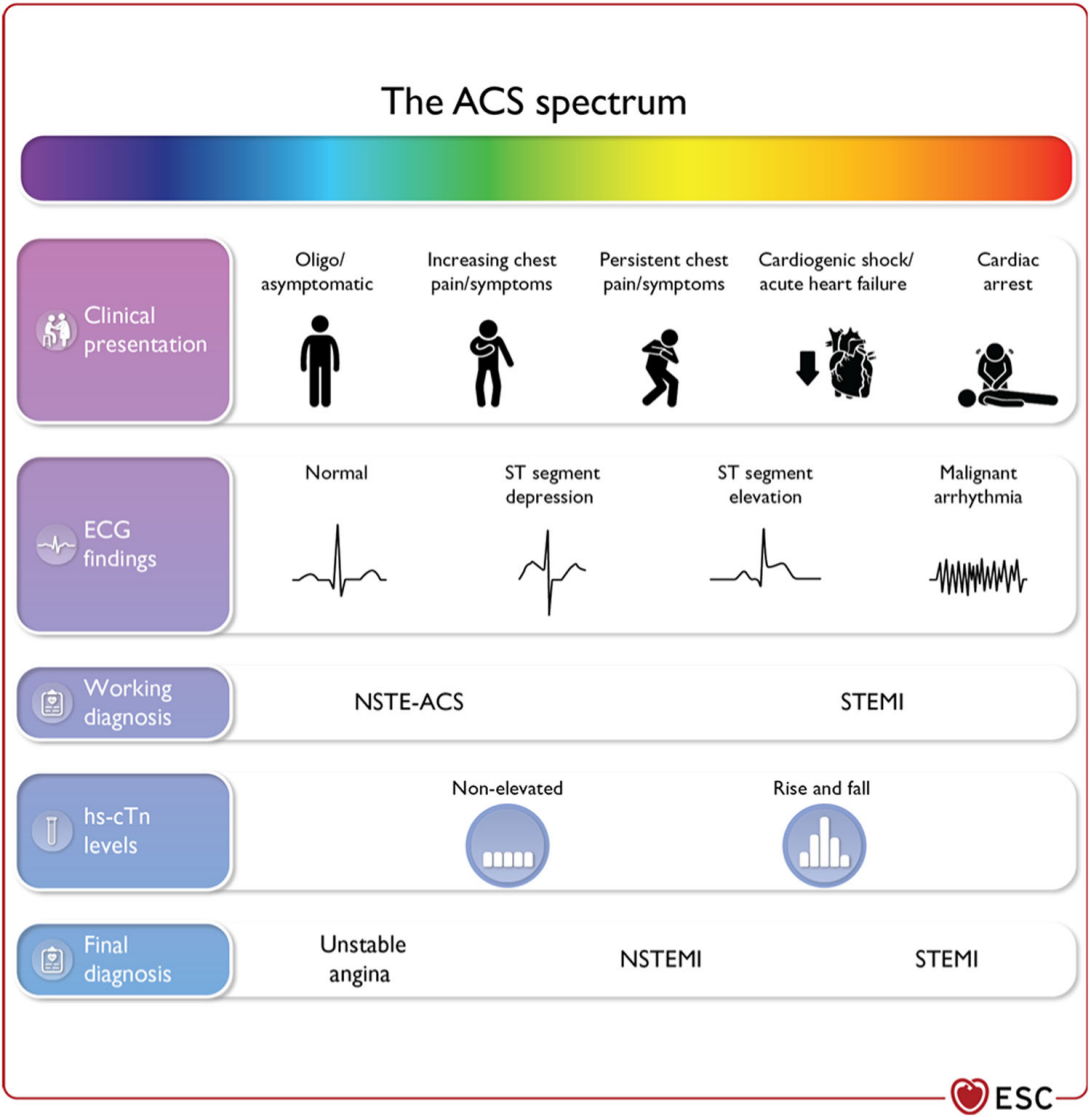
The spectrum of clinical presentations, electrocardiographic findings, and high‐sensitivity cardiac troponin levels in patients with acute coronary syndrome.

The guidelines (Figure 1) graphically explore the spectrum of clinical presentations such that the patient may initially have had chest pain, but at presentation either has minimal or no symptoms; to the patient with increasing chest pain or other symptoms; to the patient with persistent chest pain or symptoms; to the patient with cardiogenic shock or acute heart failure; and finally, the patient who presents with a cardiac arrest. The ECG may be normal at presentation, may have ST segment depression as potentially an NSTEMI, or may have ST segment elevation leading to the immediate diagnosis of STEMI. If the high‐sensitivity troponin [3] is not elevated, the resultant diagnosis is unstable angina, but the characteristic rise and fall of high‐sensitivity troponin does not differentiate between NSTEMI and STEMI.

As noted, the initial ACS assessment includes the electrocardiogram, physical examination, clinical history, vital signs, and high‐sensitivity troponin trends. An ST elevation MI is readily apparent from the electrocardiogram, but patients with NSTE‐ACS [4] should be divided into those with very high‐risk features and those without such very high‐risk features. Very high‐risk features include hemodynamic instability or cardiogenic shock; recurrent or ongoing chest pain, refractory to medical management; acute heart failure presumed secondary to ongoing myocardial ischemia; life‐threatening arrhythmias or cardiac arrest after presentation; mechanical complications [5]; or recurrent dynamic electrocardiographic changes suggestive of ischemia.

Patients with STEMI require immediate angiography and percutaneous coronary intervention (PCI) as appropriate (or thrombolysis if timely PCI is not feasible), as do the NSTE‐ACS patients with very high‐risk features. For NSTE‐ACS without very high‐risk features, angiography should be considered within the initial 24 h [6, 7]. The results of angiography determine the need for PCI [8] or coronary artery bypass grafting (CABG). Then warranted is the consideration of long‐term medical therapy and lifestyle measures, with an emphasis on smoking cessation.

## 4 Delays in Care

The guidelines explore the potential delays for patients presenting with STEMI. These include patient self‐presentation delay or emergency medical system (EMS) delay and systems of care delay within the receiving medical center [9], all adding to the total ischemic time. Analysis of these components may improve the delivery of care for patients with STEMI and will involve patient education for prompt presentation, the EMS system efficiency, the emergency room contact in the hospital, and transfer to the cardiac catheterization laboratory, ideally in under 90 min [10]. The guidelines further detail the recommendations for patients presenting to a non‐PCI center, scheduling transfer to an appropriate more intensive level facility.

## 5 Antithrombotic Therapy

The guidelines further delineate the antithrombotic therapy regimens that are appropriate for patients with and without indications for oral anticoagulation and present antiplatelet drug strategies designed to reduce bleeding risk in the first year after an ACS [11]. For example, for patients with very high bleeding risk [12], 1 month of dual antiplatelet therapy (DAPT—P2Y12 inhibitor and aspirin) may be feasible (IIb, B). In those with a lower bleeding risk, 3 months of such therapy may be considered. In general, 6 months of DAPT is reasonable [13, 14, 15] (IIa, A), although optimal is 1 year of DAPT followed by aspirin monotherapy or P2Y12 inhibitor monotherapy, with the latter preferred [16, 17]. An alternative DAPT de‐escalation strategy [18, 19] involves a change from aspirin plus prasugrel or aspirin plus ticagrelor to aspirin plus clopidogrel. In patients who require oral anticoagulation, NOAC monotherapy is a Class 1 indication after a year of DAPT.

## 6 Long‐Term Treatment

The long‐term management after an ACS [20] has three treatment goals: to support healthy lifestyle choices, to continue optimal pharmacologic [21] and cardioprotective treatments, and to reach and sustain risk factor treatment targets. Healthy lifestyle choices include smoking cessation, healthy diet [22], regular exercise, healthy weight, and psychosocial management. Optimal pharmacological and cardioprotective treatments include antithrombotic therapy, lipid‐lowering therapy [23, 24], annual influenza vaccination [25], and drug adherence and persistence. The attainment and maintenance of treatment targets include a systolic blood pressure of less than 130 mmHg and a diastolic blood pressure under 80 mmHg as tolerated, LDL‐C below 55 mg/dL, and a HgbA1c less than 7% [26, 27].

## 7 Person‐Centered Approach

A person‐centered approach to the ACS journey includes considering the physical and psychosocial needs of the patient at every stage [28]. Before the ACS, all risk factors should have been considered, medical history and prior medications should have been established, and there should have been consideration of psychosocial factors. Hospital admission should entail individualized care at triage, a person‐centered clinical assessment, and the employment of shared decision‐making [29]. Preparing for discharge requires explanations regarding long‐term treatment, education about lifestyle modification, and consideration of mental and emotional health. Important are patient expectations. ACS patients expect their ACS symptoms to be recognized; their physical, mental, and emotional well‐being to be considered; and that there be consideration of support for their family and carers. They further expect high quality [30, 31], effective, and safe care to be delivered by professionals; clear and comprehensive information to be delivered [32]; and attention to both their physical and environmental needs. This involves the right care at the right times; shared‐decision making and respect for patient preferences; and a clean and safe hospital environment.

## 8 New Recommendations

A number of new recommendations (subsequent to the prior guidelines) deserve emphasis. For patients who stop DAPT to undergo CABG, resumption of DAPT after surgery should be continued for a total of at least 12 months (I, C). P2Y12 inhibitor monotherapy may be an alternative to aspirin monotherapy for long‐term treatment [15] (IIb, A). In patients requiring oral anticoagulation, after antiplatelet therapies plus anticoagulation for 6 months, continuing only oral anticoagulants may be considered (IIb, B). There is emphasis that de‐escalation of antiplatelet therapy in the first 30 days after an ACS event is not recommended (III, B).

In patients with spontaneous coronary artery dissection (SCAD) [33], PCI is recommended only for patients with ongoing myocardial ischemia, a large area of myocardium in jeopardy, and reduced antegrade flow (I, C). For patients with multiple vessel disease presenting in cardiogenic shock, staged PCI of the non‐infarct‐related artery (non‐IRA) should be considered (IIa, C). However, in hemodynamically stable STEMI patients, PCI of the non‐IRA (culprit artery) should be considered, depending on angiographic severity [34] (I, B).

Regarding ACS complications, if high degree AV block does not resolve within 5 days following MI, implantation of a permanent pacemaker is recommended (I, C). With high clinical suspicion of an LV thrombus [35, 36] (IIa, C) and an equivocal echocardiographic image, cardiac magnetic resonance imaging should be considered.

## 9 Recommendations Regarding Comorbidities

A number of recommendations address patients with the comorbidity of cancer [37, 38]. In cancer patients with high‐risk ACS who have an expected survival of 6 months or more, an invasive strategy is recommended (I, B). Temporary interruption of the cancer therapy is recommended when the cancer therapy is suspected to be a contributing cause of the ACS (I, C). A conservative noninvasive strategy should be considered in patients with a poor cancer prognosis, such as a life expectancy under 6 months and/or those with very high bleeding risk (IIa, C). For cancer patients with a platelet count below 10 000 μg/L, aspirin is not recommended (III, C); clopidogrel is not recommended if the platelet count is below 30 000 μg/L (III, C) and prasugrel or ticagrelor is not recommended with a platelet count below 50 000 μg/L (III, C).

Intensive implementation of lipid‐lowering therapy during ACS hospitalization is recommended for patients on lesser lipid‐lowering therapy before admission (I, C).

## 10 Patient‐Centered Care

Patient‐centered care involves assessing and adhering to individual patient preferences, needs, and beliefs so that patient values inform clinical decisions (I, B). ACS patients must be included in decision‐making and informed about the risk of adverse events, radiation exposure, and alternative options. It is suggested that decision aids can facilitate this discussion (I, B). Recommendation is made to assess symptoms using methods that help patients describe their experience and use the “teach back” technique for decision support during the securing of informed consent (IIa, B). Patient discharge information should be provided in both written and verbal formats before discharge and adequate preparation and education for discharge using “teach back” techniques and formal motivational interviewing should provide information and check for understanding (IIa, B). Assessment of mental well‐being using a validated tool and a psychological referral when appropriate should be considered (IIa, B).

## 11 Summary

In summary, the highlights of the ECS‐ACS Guidelines include the concept that ACS encompasses a spectrum from unstable angina to NSTEMI to STEMI. The serial approach involves thinking A.C.S. at initial assessment; thinking invasive management; thinking antithrombotic therapy; thinking revascularization; and thinking secondary prevention.

Items in parentheses indicate class of recommendation and level of evidence.

## Conflicts of Interest

The author declares no conflicts of interest.

## Data Availability

Data sharing not applicable to this article as no datasets were generated or analyzed during the current study.
